# Birth-to-childhood tracking of linear growth and weight gain in the MINA-Brazil Study

**DOI:** 10.11606/s1518-8787.2023057005628

**Published:** 2024-02-01

**Authors:** Bárbara Hatzlhoffer Lourenço, Caroline Zani Rodrigues, Ana Alice de Araújo Damasceno, Marly Augusto Cardoso, Marcia C. Castro

**Affiliations:** I Universidade de São Paulo Faculdade de Saúde Pública Departamento de Nutrição São Paulo SP Brasil Universidade de São Paulo. Faculdade de Saúde Pública. Departamento de Nutrição. São Paulo, SP, Brasil; II Universidade de São Paulo Faculdade de Saúde Pública São Paulo SP Brasil Universidade de São Paulo. Faculdade de Saúde Pública. Programa de Pós-Graduação Nutrição em Saúde Pública. São Paulo, SP, Brasil; III Universidade Federal do Acre Cruzeiro do Sul AC Brasil Universidade Federal do Acre. Centro Multidisciplinar. Cruzeiro do Sul, AC, Brasil; IV Harvard TH Chan School of Public Health Department of Global Health and Population Boston MA United States of America Harvard TH Chan School of Public Health. Department of Global Health and Population. Boston, MA, United States of America

**Keywords:** Child, Nutritional Status, Growth, Weight Gain, Socioeconomic Factors, Birth Cohort

## Abstract

**OBJECTIVE:**

To investigate birth-to-childhood tracking of linear growth and weight gain across the distribution of length/height and weight for age z-scores and according to household wealth.

**METHODS:**

Data from 614 children from the MINA-Brazil Study with repeated anthropometric measurements at birth and up to age five years were used. Z-scores were calculated for length/height (HAZ) and weight (WAZ) according to international standards. Birth-to-childhood tracking was separately estimated using quantile regression models for HAZ and WAZ, extracting coefficients and 95% confidence intervals (95%CI) at the 25^th^, 50^th^, and 75^th^ quantiles. In a subgroup analysis, we estimated tracking between birth and age two years, and between ages two and five years. To investigate disparities in tracking, interaction terms between household wealth indexes (at birth and age five years) and newborn size z-scores were included in the models.

**RESULTS:**

Tracking coefficients were significant and had similar magnitude across the distribution of anthropometric indices at age five years (HAZ, 50^th^ quantile: 0.23, 95%CI: 0.11 to 0.35; WAZ, 50^th^ quantile: 0.31, 95%CI: 0.19 to 0.43). Greater tracking was observed between ages two and five years, with coefficients above 0.82. Significantly higher tracking of linear growth was observed among children from wealthier households, both at birth, at the lower bounds of HAZ distribution (25^th^ quantile: 0.30, 95%CI: 0.13 to 0.56), and during childhood, in the entire HAZ distribution at five years. For weight gain, stronger tracking was observed at the upper bounds of WAZ distribution at age five years among children from wealthier households at birth (75^th^ quantile: 0.59, 95%CI: 0.35 to 0.83) and during childhood (75^th^ quantile: 0.54, 95%CI: 0.15 to 0.93).

**CONCLUSION:**

There was significant tracking of HAZ and WAZ since birth, with indication of substantial stability of nutritional status between ages two and five years. Differential tracking according to household wealth should be considered for planning early interventions for preventing malnutrition.

## INTRODUCTION

As child growth stands as an important indicator of population health status, the occurrence of malnutrition for up to age five years is concerning. In Latin America and the Caribbean, stunting and overweight affected 11.5 and 8.6% of children in this age group as of 2022, respectively^1^. According to the Brazilian National Survey on Child Nutrition (ENANI-2019)^2^, the corresponding figures countrywide were 7.0 (95%CI: 6.0 to 7.9) and 10.1% (95%CI: 9.0 to 11.1). Despite reductions in disparities over time, parameters of linear growth and weight gain were still lower for children from the Northern region of Brazil, in comparison to the other macroregions^3^. Notably, in lower-resource settings, the prevalence of nutritional deficits should be considered in light of potential decreases in the entire distribution of anthropometric indices, characterizing suboptimal growth as a whole-population condition^4^.

Evidence indicates that the promotion of healthy child growth patterns starts with better fetal growth conditions^5^. Pooled analysis of 33 cohort studies in low- and middle-income countries underlined the favorable effects of population-level improvements in size at birth for the nutritional status of infants and young children^6^. For instance, a shift to birth length ≥ 50 cm in two-thirds of the population was associated with a mean difference of +0.40 in length-for-age z-score at age two years^6^. Global data have found that growth faltering accumulates particularly up to this age, with negative repercussions throughout life^7,8^.

Ensuring the desirable medium- to long-term benefits of early preventive actions against malnutrition is contingent on the growth course over the years^9^. Tracking coefficients may quantify the contribution of an earlier measurement, representing the development attained during a sensitive period, to an individual's trajectory at a later point in time. For children, therefore, connecting the assessment of antenatal and postnatal growth seems critical^10^.

In this sense, there are some key aspects to be pondered. First, given that growth is a continuous, time-dependent process, it would be optimal to evaluate tracking of anthropometric measures by age and based on a common prescriptive approach^11,12^. Second, evidence on growth tracking usually relies on average coefficients^9^ but, if present, variations in the magnitude of tracking at the extremes of the distribution of length/height and weight could be relevant for targeting priority groups since infancy . Finally, it is known that socioeconomic inequalities, especially within-country disparities, hinder progress in improving the nutritional status of populations^13^. Thus, it is also crucial to explore the extent to which tracking relative to optimal growth standards is affected by different levels of local wealth.

This study aimed to investigate the tracking of linear growth and weight gain from birth to age five years among Amazonian children followed up in the MINA-Brazil Study across the distribution of these anthropometric measures. We additionally explored whether there is differential tracking according to household wealth and performed subgroup analyses of tracking from birth to age two years and from age two to age five years in order to examine sensitive intervals to the occurrence of malnutrition.

## METHODS

### Study Design and Participants

The Maternal and Child Health and Nutrition in Acre, Brazil (MINA-Brazil Study), is a population-based birth cohort in the municipality of Cruzeiro do Sul, in the Brazilian Amazon area (estimated population: 81,519). The setting is endemic for malaria and characterized by disparities in socioeconomic and health access indicators. The cohort profile of the MINA-Brazil Study has been previously described in detail elsewhere^14^.

**Figure 1:**
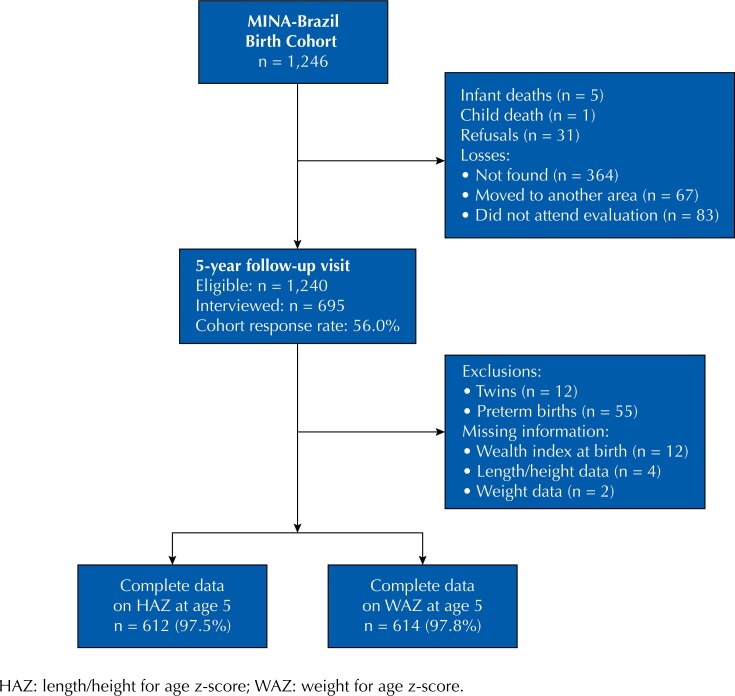
Flowchart of participants in the MINA-Brazil Study included in the analysis.

Baseline was established from July 2015 to June 2016. All birth-related admissions were screened by the study team at *Hospital da Mulher e da Criança do Vale do Juruá* (Juruá Valley Women and Children's Hospital), in which 96% of all deliveries take place. Out of 1,881 admissions, there were 112 abortions and 16 stillbirths. From a total of 1,753 live births, 184 mothers refused to participate, 18 were not assessed before hospital discharge, and 305 mother-baby pairs were living in remote rural areas. Therefore, 1,246 participants were eligible for follow-up visits that were planned up to age five years. In this period, six infant and child deaths were recorded. For this analysis, the flow diagram is shown in Figure 1.

During hospital stay, medical records were reviewed and interviews were conducted with participants within the first 12 hours after childbirth^14^. For the five-year follow-up visit, data collection was carried out in two rounds at a major primary health care unit in the urban area of Cruzeiro do Sul: in January 2021, focusing on children who were born from July to December 2015, and in June/July 2021, for those who were born from January to June 2016. A total of 695 participants (56% of 1,240 eligible children) were assessed. Despite presenting comparable perinatal characteristics, these children were from significantly wealthier households and more frequently born to mothers with > 9 years of schooling, when compared to those who were lost to follow-up (n = 514)^15^. In this study, we excluded children born at < 37 gestational weeks and twins.

Written informed consent was obtained from all participants at birth and at the five-year follow-up visit. Legal guardians provided consent when mothers were aged < 18 years. All study procedures were approved by the Institutional Review Board of the School of Public Health, Universidade de São Paulo (protocols 872.613/2014, 2.358.129/2017).

### Anthropometric Measurements

Data on the child's birth length (cm) and weight (kg) were collected from medical records at the maternity hospital, along with sex and gestational age. At the beginning of baseline, research assistants provided training on standardized anthropometric procedures for measuring birth length and weight to hospital staff involved in newborn care, including nurses, nursing assistants, and technicians from the obstetric and surgical centers. Birth length was measured with an inextensible measuring tape (SECA, model 218) and birth weight was assessed using a portable Toledo Júnior digital scale to the nearest 0.005 kg. The accuracy of the scale was regularly checked by the study team^14^. Gestational age as recorded in medical records presented good agreement with the information confirmed with ultrasound scans performed by the study team during the antenatal period in a subsample of participants in the MINA-Brazil Study^16^. Birth length and weight z-scores according to gestational age and sex were calculated considering the INTERGROWTH-21^st^ Project international standards^12^.

At the five-year follow-up visit, trained research assistants obtained anthropometric measurements in duplicates from children in light clothing and without shoes, according to standardized procedures^17^. Child's height (m) was assessed using a portable stadiometer (Alturexata^®^) to the nearest millimeter. Participants were positioned in the middle of the stadiometer in a vertical standing position, with their head, shoulders, buttocks, and heels properly aligned. Child's weight (kg) was obtained with an electronic scale (model UM061, Tanita Corporation) to the nearest 100 g. Measurements were allowed to vary up to 0.2 cm and 100 g, respectively, and mean values were used for the calculation of sex-specific height for age (HAZ) and weight for age (WAZ) z-scores using the WHO Growth Standards^11^.

For a subgroup analysis, we considered HAZ and WAZ at age two years among children who were followed-up at age five years. At the two-year follow-up, child's recumbent length was ascertained with a horizontal measuring board laid on a flat and firm surface; procedures for assessing child's weight involved subtracting maternal weight from a combined weight measure of the mother and the child. Quality control and precision standards of anthropometric measurements were identical to those described above. At all ages, children were classified according to < −2 z-scores of attained length/height and weight. For weight, we also identified the proportion of participants at > +2 z-scores.

### Socioeconomic Characteristics

A structured questionnaire gathered information on the ownership of the following household assets (yes or no) at birth and in childhood: television, sound equipment, computer, DVD player, access to the internet, paid TV, gas stove, refrigerator, blender, electric iron, microwave, upholstered sofa set, landline telephone, cell phone, bicycle, motorcycle, car, and ownership of land/farm and cattle. For each assessment, principal component analysis was employed to generate an index based on the sum of standardized scores for the items^18^. The resulting household wealth index was divided into tertiles (highest, intermediate, lowest); the third tertile corresponded to the wealthiest households and the first tertile to the poorest households.

At the five-year follow-up visit, household food insecurity referring to the three months prior to the interview was assessed using the validated short version of the Brazilian Food Insecurity Scale^19^. In total, five questions investigated whether there was a concern for shortness of food in the household, as well as whether the diet was not healthy and varied or if it was necessary to skip meals or to reduce the amount of food eaten due to insufficient resources in the household. A positive answer to any of the questions was considered indicative of food insecurity.

### Statistical Analysis

Characteristics at birth and in childhood among participants were described as medians and interquartile ranges (IQR) or proportions. From birth to age five years, median values and proportions of interest of anthropometric indices were compared using the Wilcoxon test and the McNemar test (for paired data).

We estimated overall birth-to-childhood tracking of linear growth and weight gain using quantile regression models separately for HAZ and WAZ. Measurements at age five years were regressed on z-scores of birth length and weight for gestational age, respectively. Coefficients and 95% confidence intervals (95%CI) were extracted at the 25^th^, 50^th^, and 75^th^ quantiles of the HAZ and WAZ distributions at age five years to explore the pattern of tracking across distributions. Models were adjusted for the child's exact age at the five-year follow-up visit.

All these z-scores were derived from international standards that shared a prescriptive approach for both intrauterine and child growth in the development of the respective curves^11,12^. Coefficients from the quantile regressions may be interpreted as correlation coefficients^20^ or the tracking exhibited between the pair of each of the anthropometric indices at different quantiles of HAZ and WAZ at age five years. Although z-scores were sex-specific, we additionally sought evidence for differential tracking by including an interaction term of sex with z-scores of birth length and weight.

In a subgroup analysis, we focused on participants who were followed-up at age five years and had available HAZ and WAZ at age two years (90% of the total analytical sample, which had similar sociodemographic and perinatal characteristics). Among these children, we further estimated tracking coefficients of linear growth and weight gain between birth and two years and between two and five years of age. Estimates (95%CI) were also extracted at the 25^th^, 50^th^, and 75^th^ quantiles of the HAZ and WAZ distributions, with adjustment for the participant's age, respectively at ages two and five years. We used scatter plots to provide a visual comparison of tracking strength among these age intervals.

Finally, to investigate disparities in birth-to-childhood tracking of linear growth and weight gain, we used quantile regression models at the 25^th^, 50^th^, and 75^th^ quantiles including socioeconomic variables of interest and their interaction term with z-scores of birth length and weight. In total, three socioeconomic variables were considered in distinct models: household wealth at birth (tertiles); household wealth in childhood (tertiles); and food insecurity in childhood (yes or no). The highest level of wealth and absence of food insecurity were considered the reference categories. Stratified analyses were subsequently performed to report HAZ and WAZ tracking coefficients (95%CI) by categories of socioeconomic variables. P-values for difference refer to the interaction terms. Statistical significance was set at 5%. All analyses were carried out on Stata, version 15.1 (StataCorp, College Station, TX).

## RESULTS

Out of 695 children interviewed at the five-year follow-up visit, 628 were eligible for the analysis, with complete information on outcomes and exposures of interest available for over 97.5% of them. Children with complete data were not significantly different from those who were followed-up and complied with the eligibility criteria for this analysis. At birth, median (IQR) z-scores for gestational age were 0.06 (−0.58 to 0.86) for length and 0.18 (−0.48 to 0.87) for weight (Table 1). During follow-up, there was no significant variation in HAZ (age 5, median: 0.06, IQR = −0.67 to 0.78; p = 0.27) and WAZ (age 5, median = 0.17, IQR = −0.59 to 1.10; p = 0.12). The occurrence of stunting remained stable (birth, 2.1% *versus* age five years, 2.3%; p = 0.85), but significant increases were observed for underweight (birth, 0.6% *versus* age five years, 2.6%; p = 0.008) and overweight (birth, 3.6% *versus* age five years, 12.7%; p < 0.001).

**Table 1:** Characteristics at birth and in childhood among participants of the MINA-Brazil Study.

Characteristic		n	Median (IQR) or %
Sex		
	Female	297	48.4
	Male	317	51.6
Gestational age at birth (weeks)		614	39.7 (39, 40)
Birth length (cm)		613	49 (48, 51)
Birth length for gestational age (z-score)^a^		613	0.06 (-0.58 to 0.86)
Birth length classification^a^		
	< −2 z-scores	600	97.9
	≥ -2 z-scores	13	2.1
Birth weight (kg)		614	3.31 (3.05 to 3.62)
Birth weight for gestational age (z-score)^a^		614	0.18 (-0.48 to 0.87)
Birth weight classification^a^			
	< -2 z-scores	4	0.6
	≥ -2 and ≤+2 z-scores	588	95.8
	> +2 z-scores	22	3.6
Household wealth at birth			
	Highest	286	46.6
	Intermediate	230	37.4
	Lowest	98	16.0
Age in childhood (years)		614	5.2 (5.1 to 5.4)
Height in childhood (m)		613	1.12 (1.08 to 1.15)
Height for age in childhood (z-score)^b^		613	0.06 (-0.67 to 0.78)
Height classification in childhood^b^			
	< -2 z-scores	599	97.7
	≥ -2 z-scores	14	2.3
Weight in childhood (kg)		614	19.4 (17.4 to 22.2)
	Weight for age in childhood (z-score)^b^	614	0.17 (-0.59 to 1.10)
Weight classification in childhood^b^			
	< -2 z-scores	16	2.6
	≥ -2 and ≤ +2 z-scores	520	84.7
	> +2 z-scores	78	12.7
Household wealth in childhood			
	Highest	206	33.5
	Intermediate	205	33.4
	Lowest	203	33.1
Food insecurity in childhood			
	No	287	46.7
	Yes	327	53.3

Note: number of observations may vary due to missing observations.

a Calculated according to the INTERGROWTH-21^st^ Project standards for newborn size by gestational age and sex.

b Calculated according to the WHO Child Growth Standards by age and sex.

As shown in Table 2, birth-to-childhood tracking of linear growth was significant, with similar magnitude across the HAZ distribution at age five years (e.g., 50^th^ quantile = 0.23, 95%CI: 0.11 to 0.35). Overall, significant tracking of weight gain from birth to five years also had an equivalent pattern across the WAZ distribution, reaching 0.38 (95%CI: 0.19 to 0.56) at the 75^th^ quantile. There was no evidence for differential tracking according to sex (p for interaction > 0.30 for HAZ and WAZ).

**Table 2:** Overall birth-to-childhood tracking of linear growth and weight gain in the MINA-Brazil Study.

Characteristic		n	Tracking coefficients and 95% confidence intervals across the distribution^a^,^b^		
			Quantile 0.25	Quantile 0.50	Quantile 0.75
Birth to 5 years					
	HAZ	612	0.17 (0.07 to 0.27)	0.23 (0.11 to 0.35)	0.23 (0.13 to 0.33)
	WAZ	614	0.33 (0.21 to 0.45)	0.31 (0.19 to 0.43)	0.38 (0.19 to 0.56)
Birth to 2 years					
	HAZ	551	0.23 (0.12 to 0.34)	0.20 (0.10 to 0.29)	0.28 (0.16 to 0.40)
	WAZ	550	0.41 (0.29 to 0.53)	0.32 (0.21 to 0.43)	0.35 (0.21 to 0.48)
2 to 5 years				
	HAZ	550	0.82 (0.75 to 0.89)	0.88 (0.82 to 0.94)	0.89 (0.84 to 0.94)
	WAZ	550	0.86 (0.80 to 0.92)	0.96 (0.89 to 1.03)	1.10 (1.00 to 1.20)

HAZ: length/height for age z-score; WAZ: weight for age z-score.

a Anthropometric indices were calculated at birth according to the INTERGROWTH-21^st^ Project standards for newborn size by gestational age and sex and at ages two and five years according to the WHO Child Growth Standards by age and sex.

b Tracking was estimated using quantile regression models separately for HAZ and WAZ. Z-scores at a later age were regressed on those from the earlier age, extracting coefficients and 95% confidence intervals at the 25^th^, 50^th^, and 75^th^ quantiles.

**Figure 2:**
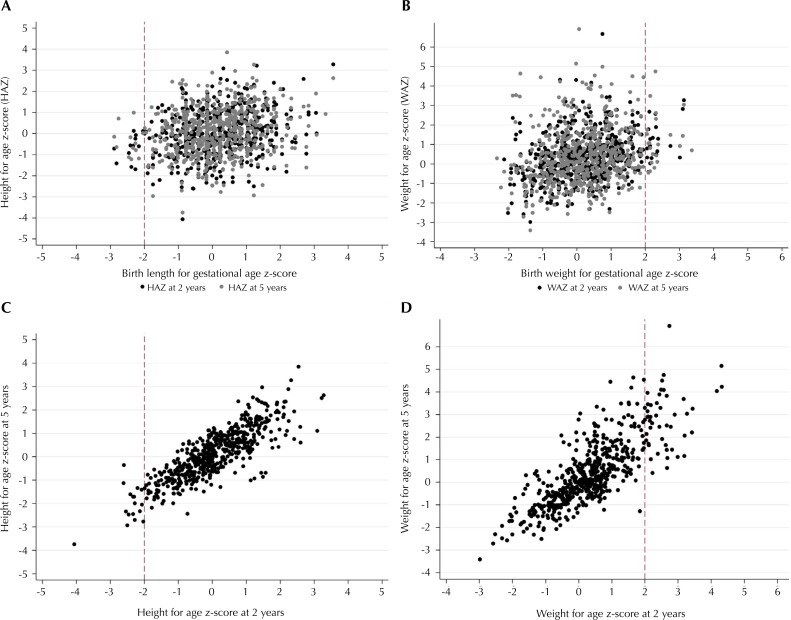
Dispersion of length/height and weight z-scores at birth and ages two and five years years among participants in the MINA-Brazil Study.

Note: Panels A and B: measurements at ages two (black dots) and five years (gray dots) in relation to measurements at birth. Panels C and D: measurements at age five years in relation to measurements at age two years (black dots). Red dashed line indicates length/height z-score < -2 (Panels A and C) and weight z-score > +2 (Panels B and D). Anthropometric indices were calculated at birth according to the INTERGROWTH-21^st^ Project standards for newborn size by gestational age and sex to and at ages two and five years according to the WHO Child Growth Standards by age and sex.

In comparison to the period from birth to five years, all tracking coefficients up to two years of age had comparable magnitude (Figures 2A and 2B), without variation across quantiles (Table 2). However, markedly greater tracking was observed from two to five years of age at all quantiles for both linear growth (50^th^ quantile: 0.88, 95%CI: 0.82 to 0.94) and weight gain (50^th^ quantile: 0.96, 95%CI: 0.89 to 1.03), as illustrated in Figures 2C and 2D.

There were disparities in the tracking of linear growth according to socioeconomic variables (Table 3). Significantly greater tracking was observed at the lower bounds of HAZ distribution at age five years among children exposed to higher household wealth at birth (25^th^ quantile: 0.30, 95%CI: 0.13 to 0.56; 50^th^ quantile: 0.38, 95%CI: 0.21 to 0.55; p-value for difference < 0.05). At all quantiles of HAZ distribution at age five years, tracking of linear growth since birth was stronger for children from the wealthiest households as measured in childhood relative to those from the poorest households, among whom coefficients were non-significant (e.g., 50^th^ quantile: 0.37, 95%CI: 0.21 to 0.53 *versus* 0.11, 95%CI: −0.07 to 0.28; p-value for difference = 0.012). At the 75^th^ quantile of HAZ distribution at age five years, there was a marginal difference in tracking of linear growth according to household food insecurity in childhood (not exposed: 0.32, 95%CI: 0.16 to 0.48; exposed: 0.12, 95%CI: 0.00 to 0.24; p-value for difference = 0.047).

**Table 3:** Birth-to-childhood tracking of linear growth and weight gain according to socioeconomic groups in the MINA-Brazil Study.

Characteristic			Tracking coefficients across the distribution at five years^a^					
			Quantile 0.25		Quantile 0.50		Quantile 0.75	
			Coef. (95%CI)^b^	p diff^c^	Coef. (95%CI)^b^	p diff^c^	Coef. (95%CI)^b^	p diff^c^
Height-for-age z-score (HAZ)								
	Household wealth at birth							
		Highest	0.30 (0.13 to 0.56)	-	0.38 (0.21 to 0.55)	-	0.30 (0.16 to 0.45)	-
		Intermediate	0.04 (−0.10 to 0.18)	0.007	0.12 (−0.02 to 0.26)	0.022	0.10 (−0.11 to 0.31)	0.198
		Lowest	0.11 (−0.19 to 0.41)	0.141	0.16 (−0.14 to 0.46)	0.103	0.12 (−0.09 to 0.32)	0.255
	Household wealth in childhood							
		Highest	0.41 (0.18 to 0.64)	-	0.37 (0.21 to 0.53)	-	0.35 (0.16 to 0.53)	-
		Intermediate	0.04 (−0.14 to 0.21)	0.003	0.22 (0.03 to 0.40)	0.194	0.20 (−0.02 to 0.41)	0.189
		Lowest	0.09 (−0.10 to 0.27)	0.010	0.11 (−0.07 to 0.28)	0.012	0.10 (−0.04 to 0.25)	0.014
	Food insecurity in childhood							
		No	0.13 (−0.03 to 0.30)	-	0.34 (0.18 to 0.49)	-	0.32 (0.16 to 0.48)	-
		Yes	0.13 (0.01 to 0.25)	0.689	0.20 (0.06 to 0.35)	0.387	0.12 (0.00 to 0.24)	0.047
Weight-for-age z-score (WAZ)								
	Household wealth at birth							
		Highest	0.42 (0.23 to 0.62)	-	0.44 (0.25 to 0.63)	-	0.59 (0.35 to 0.83)	-
		Intermediate	0.33 (0.16 to 0.50)	0.550	0.20 (0.00 to 0.40)	0.019	0.03 (−0.29 to 0.34)	0.003
		Lowest	0.18 (−0.11 to 0.46)	0.192	0.19 (−0.12 to 0.51)	0.032	0.40 (−0.10 to 0.89)	0.112
	Household wealth in childhood							
		Highest	0.47 (0.24 to 0.70)	-	0.67 (0.46 to 0.86)	-	0.54 (0.15 to 0.93)	-
		Intermediate	0.28 (0.08 to 0.47)	0.192	0.30 (0.11 to 0.49)	0.013	0.35 (0.05 to 0.66)	0.308
		Lowest	0.27 (0.08 to 0.46)	0.186	0.14 (−0.08 to 0.35)	<0.001	0.08 (−0.25 to 0.41)	0.031
	Food insecurity in childhood							
		No	0.27 (0.07 to 0.47)	-	0.43 (0.22 to 0.65)	-	0.52 (0.25 to 0.79)	-
		Yes	0.40 (0.26 to 0.54)	0.348	0.30 (0.16 to 0.45)	0.241	0.24 (0.05 to 0.44)	0.060

HAZ: length/height for age z-score; WAZ: weight for age z-score; Coef.: coefficient; 95%CI: 95% confidence interval; p diff: p-value for difference.

a Anthropometric indices were calculated at birth according to the INTERGROWTH-21^st^ Project standards for newborn size by gestational age and sex to and at age five years according to the WHO Child Growth Standards by age and sex.

b Tracking was separately estimated using quantile regression models for HAZ and WAZ. Z-scores at age five years were regressed on those from birth to extracting coefficients and 95% confidence intervals at the 25^th^ to 50^th^ to and 75^th^ quantiles

c To investigate disparities to variables of household wealth and food insecurity were interacted with z-scores at birth. P-values for difference refer to the interaction terms. Stratified analyses were performed to report HAZ and WAZ tracking coefficients by categories of socioeconomic variables.

For weight gain (Table 3), stronger tracking was observed at the 50^th^ and 75^th^ quantiles of WAZ distribution at age five years among children from the wealthiest households at birth and during childhood in comparison to participants from households at intermediate and lowest wealth status. Median birth-to-childhood tracking of weight gain was 0.44 (95%CI: 0.25 to 0.63) among participants from better-off households at birth (relative to 0.19, 95%CI: −0.12 to 0.51 among those from the poorest households at birth, p-value for difference = 0.032) and reached 0.67 (95%CI: 0.46 to 0.86) for wealthiest children in childhood (relative to 0.14, 95%CI: −0.08 to 0.35 among those from the poorest households as measured in childhood; p-value for difference < 0.001). There was no evidence for differences in the tracking of weight gain since birth across the distribution of WAZ according to food insecurity in at age five years.

## DISCUSSION

Using prospectively collected data from children aged up to five years participating in the MINA-Brazil Study, we found significant and positive birth-to-childhood tracking of linear growth and weight gain, with coefficients of similar magnitude across the distribution of the anthropometric indices. Tracking of nutritional status was stronger in the period between ages two and five years. Also, there was differential tracking according to exposure to distinct levels of household wealth.

In our study population, average newborn size at birth and nutritional status at five years were both close to the expected median values for length/height and weight for age by international standards. We noted a slight shift toward the left for the continuous values of HAZ until age five years, as depicted by the interquartile ranges, but stunting remained low during follow-up. For child's weight, the distribution has become more spread out over time, with lower values of WAZ corresponding to the 25^th^ quantile and higher values of WAZ at the 75^th^ quantile at age five years. This was reflected in significantly higher occurrence of underweight and overweight in childhood, characterizing some of the dynamics of malnutrition in the MINA-Brazil Study.

Tracking of linear growth and weight gain was significant overall and comparable among girls and boys. Coefficients of moderate magnitude and overlapping 95%CI ranged from 0.17 to 0.39 from birth to age five years and, in a subgroup analysis, from 0.20 to 0.40 from birth to age two years. Taken together, these estimates quantify a not negligible and rather lasting influence of fetal growth conditions, as summarized by the measures of size at birth, on the entire distribution of child's anthropometry. This is in line with longitudinal findings on the importance of the gestational period in preventing growth faltering and recognizing room for additional measures early in life to contest postnatal insults to healthy height and weight accrual^6^. According to a recent review, effective measures to leverage infant and young child growth include, but are not limited to, breastfeeding and complementary feeding counseling; supplementation of multiple micronutrients; family planning; malaria control; and water, sanitation, and hygiene interventions^21^.

From age two years, tracking exhibited substantially larger coefficients across the distributions of HAZ and WAZ, above 0.82. This result connects tightly with the importance of the first 1,000 days in fostering human development. Standing as a complementary approach to tracking coefficients in exploring growth trajectories, measures of conditional body size indicated that faster linear growth and weight gain up to age two years were beneficial to adult height and schooling in low- and middle-income countries, besides protecting against some risk factors for adult chronic disease^22,23^. Simultaneously, declines in z-scores of length for age are known to peak essentially in the second year of life^24^, drawing a challenging scenario for children in lower-resource settings. Encompassing an assessment with standards for optimal growth, the estimates of the present study suggested that, in fact, the levels of nutritional status adequacy attained after the first two years will track strongly until age five years. This delimits a lower probability for spontaneous changes in the patterns of trajectories in this interval and reinforces the need for timely evidence-based interventions.

Socioeconomic disparities had distinct roles to shape the tracking of HAZ and WAZ. Regarding height at age five years, belonging to wealthier households was associated with a significantly stronger tracking since birth across the entire distribution, which implies better possibilities for retaining the positive influence of intrauterine linear growth postnatally. Also, the relationship appeared to be most relevant for children at the lower bounds of HAZ distribution, if considering wealth at birth. Given that children showing HAZ values below the expected median for their age are probably subject to worse environmental insults to growth, detaining a better socioeconomic position could be valuable. This is consistent with global meta-analyzed data indicating the concentration of stunting among children younger than five years in poor households, especially in Latin America and the Caribbean (regional pooled concentration index: -0.22, 95%CI: -0.29 to -0.15)^13^. In the combined analysis of cohorts from low- and middle-income countries, early-life poverty at the household level has also been found to negatively impact attained growth at later periods, during adolescence and adulthood^25,26^.

Referring to WAZ at age five years, stronger correlation coefficients were apparent for heavier children from better-off households. In cohorts from the Young Lives Study, the relative risk ratio for adhering to a trajectory of high probability of overweight varied from 3.1 to 5.6 in the top wealth quartiles in India, Peru, and Vietnam^25^. This was also coherent with a systematic review of studies in middle-income countries that found a majority of positive associations between socioeconomic position and fat measures of body composition among school-aged children (78% of 18 studies with girls and 63% of 9 studies with boys)^27^, as opposed to children from high-income countries, in which inverse associations emerge in several reports^20,27,28^.

The present analysis offered a quantitative surrogate of trajectories of linear growth and weight gain and standardized serial anthropometric measurements under common recommendations from international curves to originally connect the interpretation of antenatal and postnatal growth processes. Our findings highlighted the importance of devising primary programs for promoting better growth patterns since before birth, with equitable access and quality according to local socioeconomic characteristics, as household wealth incurred in differential tracking of nutritional status.

Some limitations should be outlined. First, attrition in our cohort reduced sample size for this complete case analysis. Also, it is important to note that the losses at follow-up were concentrated among children from worse socioeconomic background and selection bias may have attenuated our estimates. Second, birth variables were collected from medical records. Measurement bias should not be substantial as training on the anthropometric assessment of newborns was provided by the study team, who also regularly checked the equipment for ascertaining birth size^14^. Moreover, agreement of information on gestational age was deemed satisfactory in comparison to measures directly obtained by the study team using ultrasound scans during the antenatal period^16^. Third, information on household food insecurity was available only at age five years and this prevented us from ascertaining associations over time with tracking of linear growth and weight gain. Also, more repeated measures would be welcome for examining tracking in narrower age intervals, but we could still investigate key sensitive periods for preventing child malnutrition.

In conclusion, we found significant tracking of linear growth and weight gain from birth to age five years at a similar magnitude across the distribution of HAZ and WAZ. Substantial stability of the levels of nutritional status adequacy specifically emerged between ages two and five years. There was differential tracking according to household wealth, which should be taken into account in planning early interventions for promoting healthy growth.

## References

[1] United Nations Children's Fund, World Health Organization, World Bank Group (2023). Levels and trends in child malnutrition: key findings of the 2023 edition.

[2] Alves-Santos NH, Castro IR, Anjos LA, Lacerda EM, Normando P, Freitas MB (2021). General methodological aspects in the Brazilian National Survey on Child Nutrition (ENANI-2019): a population-based household survey. Cad Saude Publica.

[3] Universidade Federal do Rio de Janeiro (2022). Estado nutricional antropométrico da criança e da mãe: prevalência de indicadores antropométricos de crianças brasileiras menores de 5 anos de idade e suas mães biológicas.

[4] Roth DE, Krishna A, Leung M, Shi J, Bassani DG, Barros AJ (2017). Early childhood linear growth faltering in low-income and middle-income countries as a whole-population condition: analysis of 179 Demographic and Health Surveys from 64 countries (1993-2015). Lancet Glob Health.

[5] Lourenço BH, Neves PA, Cardoso MA, Castro MC, MINA-Brazil Study Group (2023). Improved estimates of foetal growth are associated with perinatal outcomes: A latent modelling approach in a population-based birth cohort. J Glob Health.

[6] Mertens A, Benjamin-Chung J, Colford JM, Coyle J, Laan MJ, Hubbard AE (2023). Causes and consequences of child growth faltering in low- and middle-income countries. Nature.

[7] Victora CG, Onis M, Hallal PC, Blössner M, Shrimpton R (2010). Worldwide timing of growth faltering: revisiting implications for interventions. Pediatrics.

[8] Black RE, Victora CG, Walker SP, Bhutta ZA, Christian P, Onis M (2013). Maternal and child undernutrition and overweight in low-income and middle-income countries. Lancet.

[9] Bayer O, Krüger H, von Kries R, Toschke AM (2011). Factors associated with tracking of BMI: a meta-regression analysis on BMI tracking. Obesity (Silver Spring).

[10] Papageorghiou AT, Kennedy SH, Salomon LJ, Altman DG, Ohuma EO, Stones W (2018). The INTERGROWTH-21st fetal growth standards: toward the global integration of pregnancy and pediatric care. Am J Obstet Gynecol.

[11] Onis M, WHO Multicentre Growth Reference Study Group (2006). WHO Child Growth Standards based on length/height, weight and age. Acta Paediatr Suppl.

[12] Villar J, Cheikh Ismail L, Victora CG, Ohuma EO, Bertino E, Altman DG (2014). International standards for newborn weight, length, and head circumference by gestational age and sex: the Newborn Cross-Sectional Study of the INTERGROWTH-21st Project. Lancet.

[13] Alao R, Nur H, Fivian E, Shankar B, Kadiyala S, Harris-Fry H (2021). Economic inequality in malnutrition: a global systematic review and meta-analysis. BMJ Glob Health.

[14] Cardoso MA, Matijasevich A, Malta MB, Lourenco BH, Gimeno SG, Ferreira MU (2020). MINA-Brazil Study Group. Cohort profile: the Maternal and Child Health and Nutrition in Acre, Brazil, birth cohort study (MINA-Brazil). BMJ Open.

[15] Ferreira MU, Giacomini I, Sato PM, Lourenço BH, Nicolete VC, Buss LF (2022). MINA-Brazil Working Group. SARS-CoV-2 seropositivity and COVID-19 among 5 years-old Amazonian children and their association with poverty and food insecurity. PLoS Negl Trop Dis.

[16] Lourenço BH, Lima DL, Vivanco E, Fernandes RB, Duarte M, Neves PAR (2020). MINA-Brazil Study Group. Agreement between antenatal gestational age by ultrasound and clinical records at birth: a prospective cohort in the Brazilian Amazon. PLoS One.

[17] World Health Organization (1995). WHO Expert Committee on Physical Status. Physical status: the use of and interpretation of anthropometry, report of a WHO expert committee.

[18] Filmer D, Pritchett LH (2001). Estimating wealth effects without expenditure data—or tears: an application to educational enrollments in states of India. Demography.

[19] Santos LP, Lindemann IL, Motta JV, Mintem G, Bender E, Gigante DP (2014). Proposal of a short-form version of the Brazilian food insecurity scale. Rev Saude Publica.

[20] Norris T, Bann D, Hardy R, Johnson W (2020). Socioeconomic inequalities in childhood-to-adulthood BMI tracking in three British birth cohorts. Int J Obes (Lond).

[21] Keats EC, Das JK, Salam RA, Lassi ZS, Imdad A, Black RE (2021). Effective interventions to address maternal and child malnutrition: an update of the evidence. Lancet Child Adolesc Health.

[22] Adair LS, Fall CH, Osmond C, Stein AD, Martorell R, Ramirez-Zea M (2013). Associations of linear growth and relative weight gain during early life with adult health and human capital in countries of low and middle income: findings from five birth cohort studies. Lancet.

[23] Norris SA, Osmond C, Gigante D, Kuzawa CW, Ramakrishnan L, Lee NR (2012). Size at birth, weight gain in infancy and childhood, and adult diabetes risk in five low- or middle-income country birth cohorts. Diabetes Care.

[24] Benjamin-Chung J, Mertens A, Colford JM, Hubbard AE, van der Laan MJ, Coyle J (2023). Early-childhood linear growth faltering in low- and middle-income countries. Nature.

[25] Schott W, Aurino E, Penny ME, Behrman JR (2019). The double burden of malnutrition among youth: trajectories and inequalities in four emerging economies. Econ Hum Biol.

[26] Victora CG, Hartwig FP, Vidaletti LP, Martorell R, Osmond C, Richter LM (2022). Effects of early-life poverty on health and human capital in children and adolescents: analyses of national surveys and birth cohort studies in LMICs. Lancet.

[27] Staatz CB, Kelly Y, Lacey RE, Blodgett JM, George A, Arnot M (2021). Socioeconomic position and body composition in childhood in high- and middle-income countries: a systematic review and narrative synthesis. Int J Obes (Lond).

[28] White PA, Awad YA, Gauvin L, Spencer NJ, McGrath JJ, Clifford SA (2022). Household income and maternal education in early childhood and risk of overweight and obesity in late childhood: findings from seven birth cohort studies in six high-income countries. Int J Obes (Lond).

