# Supplementary material for: Birth-to-childhood tracking of linear growth and weight gain in the MINA-Brazil Study
Source: Rev Saude Publica. 2024 Feb 1;57(Suppl 2):3s. doi: 10.11606/s1518-8787.2023057005628 (PMC10897963; doi:10.11606/s1518-8787.2023057005628)
Supplement: Translation

# Translation: Autocorrelação do crescimento linear e ganho de peso do nascimento à infância no Estudo MINA-Brasil

**Authors:** Bárbara Hatzlhoffer Lourenço, Caroline Zani Rodrigues, Ana Alice de Araújo Damasceno, Marly Augusto Cardoso, Marcia C. Castro

**Affiliations:** I São Paulo SP Brasil Universidade de São Paulo. Faculdade de Saúde Pública. Departamento de Nutrição. São Paulo, SP, Brasil; II São Paulo SP Brasil Universidade de São Paulo. Faculdade de Saúde Pública. Programa de Pós-Graduação Nutrição em Saúde Pública. São Paulo, SP, Brasil; III Cruzeiro do Sul AC Brasil Universidade Federal do Acre. Centro Multidisciplinar. Cruzeiro do Sul, AC, Brasil; IV Boston MA United States of America Harvard TH Chan School of Public Health. Department of Global Health and Population. Boston, MA, United States of America

**Keywords:** Criança, Estado Nutricional, Crescimento, Ganho de Peso, Fatores Socioeconômicos, Coorte de Nascimento

## Abstract

**OBJETIVO:**

Investigar a autocorrelação do crescimento linear e ganho de peso do nascimento até a infância considerando a distribuição de escores z de comprimento/estatura e peso por idade e de acordo com a riqueza domiciliar.

**MÉTODOS:**

Foram utilizados dados de 614 crianças do Estudo MINA-Brasil com medições antropométricas realizadas do nascimento até os cinco anos de idade. Escores z foram calculados para comprimento/estatura (HAZ) e peso (WAZ) seguindo padrões internacionais. A autocorrelação ou estabilidade da adequação do estado nutricional do nascimento à infância foi estimada por meio de modelos de regressão quantílica para HAZ e WAZ, separadamente, extraindo-se coeficientes e intervalos de confiança de 95% (IC95%) nos quantis 25, 50 e 75. Em uma análise de subgrupo, estimou-se a autocorrelação entre o nascimento até os dois anos de idade, e entre dois e cinco anos de idade. Para investigar disparidades na autocorrelação, termos de interação entre índices de riqueza familiar (ao nascer e aos cinco anos de idade) e escores z de tamanho do recém-nascido foram incluídos nos modelos.

**RESULTADOS:**

Os coeficientes de autocorrelação foram significantes e tiveram magnitude semelhante ao longo da distribuição dos índices antropométricos aos cinco anos de idade (HAZ, quantil 50: 0,23, IC95%: 0,11 a 0,35; WAZ, quantil 50: 0,31, IC95%: 0,19 a 0,43). Maior estabilidade do estado nutricional foi observada entre dois e cinco anos, com coeficientes acima de 0,82. Autocorrelação significantemente maior do crescimento linear foi observada entre as crianças de domicílios mais ricos, tanto ao nascer, nos limites inferiores da distribuição HAZ (quantil 25: 0,30, IC95%: 0,13 a 0,56) e durante a infância, em toda a distribuição HAZ aos cinco anos. Para o ganho de peso, observou-se uma autocorrelação mais forte nos limites superiores da distribuição do WAZ aos cinco anos de idade entre as crianças de domicílios mais ricos ao nascer (quantil 75: 0,59, IC95%: 0,35 a 0,83) e durante a infância (quantil 75: 0,54, IC95%: 0,15 a 0,93).

**CONCLUSÃO:**

Houve autocorrelação significante de HAZ e WAZ desde o nascimento, com indicação de substancial estabilidade do estado nutricional entre os dois e cinco anos de idade. A autocorrelação diferencial de acordo com a riqueza domiciliar deve ser considerada para o planejamento de intervenções precoces para prevenir a má nutrição.

## INTRODUÇÃO

Como o crescimento infantil é um importante indicador do estado de saúde da população, a má nutrição até cinco anos de idade é preocupante. Na América Latina e no Caribe, baixa estatura e sobrepeso afetaram 11,5 e 8,6% das crianças nesta faixa etária em 2022, respectivamente^1^. De acordo com o Estudo Nacional de Alimentação e Nutrição Infantil (ENANI-2019)^2^, os números correspondentes em todo o país foram de 7,0 (IC95%: 6,0 a 7,9) e 10,1% (IC95%: 9,0 a 11,1). Apesar das reduções nas disparidades ao longo do tempo, os parâmetros de crescimento linear e ganho de peso ainda foram menores para as crianças da região Norte do Brasil, em comparação com as demais macrorregiões^3^. Notadamente em contextos com menos recursos, a prevalência de déficits nutricionais deve ser considerada à luz de potenciais prejuízos na distribuição dos índices antropométricos de forma geral, caracterizando crescimento subótimo como uma condição que afeta a totalidade da população^4^.

Evidências indicam que a promoção de padrões saudáveis de crescimento infantil inicia-se com melhores condições de crescimento fetal^5^. Uma análise conjunta de 33 estudos de coorte em países de baixa e média renda destacou os efeitos favoráveis de melhorias no tamanho ao nascer em nível populacional para o estado nutricional de lactentes e crianças pequenas^6^. Por exemplo, uma mudança para comprimento ao nascer ≥ 50 cm em dois terços da população foi associada a uma diferença média de +0,40 escore z de comprimento para idade aos dois anos de idade^6^. Dados mundiais constatam que falhas no crescimento se acumulam particularmente até essa idade, com repercussões negativas ao longo da vida^7,8^.

A garantia dos benefícios desejáveis em médio e longo prazo de ações preventivas precoces contra a má nutrição depende do curso do crescimento ao longo dos anos^9^. Coeficientes de autocorrelação ou de estabilidade do estado nutricional podem quantificar a contribuição de uma medida anterior, representando o desenvolvimento alcançado durante um período sensível, para a trajetória de um indivíduo em um momento posterior. Para as crianças, portanto, relacionar a avaliação do crescimento pré-natal e pós-natal parece fundamental^10^.

Nesse sentido, alguns aspectos chave devem ser ponderados. Primeiramente, considerando que o crescimento é um processo contínuo e tempo-dependente, seria ideal avaliar a autocorrelação das medidas antropométricas por idade, baseando-se em uma abordagem prescritiva comum^11,12^. Em segundo lugar, as evidências sobre a autocorrelação do crescimento geralmente se baseiam em coeficientes médios^9^, mas, se presentes, variações na magnitude de autocorrelação nos extremos da distribuição de comprimento/altura e peso podem ser relevantes para definição de grupos prioritários desde a infância. Por último, sabe-se que desigualdades socioeconômicas, especialmente as disparidades internas de um país, dificultam o progresso na melhoria do estado nutricional das populações^13^. Assim, também é crucial explorar até que ponto a autocorrelação em relação aos padrões ideais de crescimento é afetada por diferentes níveis locais de riqueza.

O objetivo deste estudo foi investigar a autocorrelação do crescimento linear e do ganho de peso do nascimento aos cinco anos de idade, considerando a distribuição destas medidas antropométricas, em crianças da região da Amazônia Brasileira acompanhadas no Estudo MINA-Brasil. Além disso, exploramos a existência de autocorrelação diferencial de acordo com a riqueza domiciliar e realizamos análises de subgrupos para a autocorrelação do nascimento aos dois anos de idade e dos dois aos cinco anos de idade, a fim de examinar intervalos sensíveis à má nutrição.

## MÉTODOS

### Desenho do Estudo e Participantes

O Projeto Saúde e Nutrição Materno-Infantil no Acre, Brasil (MINA-Brasil) é uma coorte de nascimentos de base populacional do município de Cruzeiro do Sul, na Amazônia Brasileira (população estimada: 81.519). A região é endêmica para malária e caracterizada por disparidades nos indicadores socioeconômicos e de acesso à saúde. O perfil da coorte do Estudo MINA-Brasil está descrito em detalhes em outras publicações^14^.

A linha de base foi estabelecida de julho de 2015 a junho de 2016. Todas as internações para parto foram triadas pela equipe do estudo no Hospital da Mulher e da Criança do Vale do Juruá, no qual 96% dos partos ocorrem. Entre 1.881 internações, houve 112 abortos e 16 natimortos. De um total de 1.753 nascidos vivos, 184 mães recusaram participar, 18 não foram avaliadas antes da alta hospitalar e 305 pares mãe-bebê viviam em áreas rurais remotas. Portanto, 1.246 participantes foram elegíveis para etapas de acompanhamento planejadas até os cinco anos de idade. Nesse período, foram registrados seis óbitos neonatais e infantis. A Figura 1 mostra o fluxograma do estudo para as presentes análises.

**Figura 1:** Fluxograma dos participantes do Estudo MINA-Brasil incluídos na análise.

Durante a internação, prontuários médicos foram revisados e entrevistas foram realizadas com as participantes nas primeiras 12 horas após o parto^14^. Para o acompanhamento aos cinco anos, a coleta de dados foi realizada em duas rodadas em uma unidade básica de saúde central da zona urbana de Cruzeiro do Sul: em janeiro de 2021, com foco nas crianças nascidas de julho a dezembro de 2015, e em junho/julho de 2021, para aquelas nascidas de janeiro a junho de 2016. No total, 695 participantes (56% de 1.240 crianças elegíveis) foram avaliados. Apesar de apresentarem características perinatais comparáveis, estas crianças eram de famílias significantemente mais ricas e mais frequentemente de mães com mais de nove anos de escolaridade, quando comparadas àquelas que não acompanhadas (n = 514)^15^. Neste estudo, foram excluídas crianças nascidas com menos de 37 semanas de gestação e gêmeos.

Todas as participantes assinaram um termo de consentimento no momento de nascimento e na consulta de acompanhamento de cinco anos. Responsáveis legais assinaram o termo quando as mães tinham menos de 18 anos. Todos os procedimentos do estudo foram aprovados pelo Comitê de Ética em Pesquisa da Faculdade de Saúde Pública da Universidade de São Paulo (protocolos 872.613/2014, 2.358.129/2017).

### Medidas Antropométricas

Dados referentes ao comprimento (cm) e ao peso (kg) da criança ao nascer foram coletados dos prontuários médicos da maternidade, juntamente com o sexo e a idade gestacional. No início da linha de base, assistentes de pesquisa ofereceram treinamento sobre procedimentos antropométricos padronizados para mensuração de comprimento e peso para a equipe hospitalar envolvida na assistência ao recém-nascido, incluindo enfermeiras, auxiliares de enfermagem e técnicos dos centros obstétricos e cirúrgicos. O comprimento ao nascer foi medido com fita métrica inextensível (SECA, modelo 218) e o peso ao nascer foi avaliado em balança digital portátil da marca Toledo Júnior, com precisão de 0,005 kg. A acurácia da balança foi verificada regularmente pela equipe do estudo^14^. A idade gestacional registrada nos prontuários apresentou boa concordância com as informações confirmadas com exames ultrassonográficos realizados pela equipe do estudo durante o pré-natal em uma subamostra de participantes do Estudo MINA-Brasil^16^. Os escores z de comprimento e peso ao nascer de acordo com a idade gestacional e o sexo foram calculados considerando padrões internacionais do Projeto INTERGROWTH-21^st^^12^.

No acompanhamento aos cinco anos, assistentes de pesquisa treinados obtiveram medidas antropométricas em duplicata das crianças, em roupas leves e sem calçados, de acordo com procedimentos padronizados^17^. A estatura (m) da criança foi avaliada em estadiômetro portátil (Alturexata^®^) com precisão de 1 mm. Os participantes foram posicionados no meio do estadiômetro em posição vertical, com cabeça, ombros, nádegas e calcanhares devidamente alinhados. O peso da criança (kg) foi obtido com balança eletrônica (modelo UM061, Tanita Corporation) com precisão de 100 g. Permitiu-se variação das medidas até 0,2 cm e 100 g, respectivamente, e os valores médios foram utilizados para o cálculo dos escores z de estatura para idade (HAZ) e peso para idade (WAZ) específicos para o sexo usando os padrões de crescimento da OMS^11^.

Em uma análise de subgrupo, foram considerados HAZ e WAZ aos dois anos de idade entre as crianças que foram acompanhadas aos cinco anos de idade. No acompanhamento aos dois anos, o comprimento da criança foi aferido em um infantômetro horizontal colocado sobre uma superfície plana e firme; os procedimentos para avaliar o peso da criança consistiram em subtrair o peso materno de uma medida combinada de peso da mãe e da criança. Padrões de controle de qualidade e precisão das medidas antropométricas foram idênticos aos descritos anteriormente. Em todas as idades, as crianças foram classificadas de acordo com escores z < −2 de comprimento/estatura e peso atingidos. Para o peso, também foi identificada a proporção de participantes com escores z > +2.

### Características Socioeconômicas

Um questionário estruturado reuniu informações sobre a posse dos seguintes bens domiciliares (sim ou não) ao nascimento e na infância: televisão, equipamento de som, computador, aparelho de DVD, acesso à internet, TV por assinatura, fogão a gás, geladeira, liquidificador, ferro elétrico, micro-ondas, sofá estofado, telefone fixo, telefone celular, bicicleta, motocicleta, carro e posse de terra/fazenda e gado. Para cada avaliação, análise de componentes principais foi empregada para gerar um índice baseado na soma dos escores padronizados para os itens^18^. O índice de riqueza domiciliar resultante foi dividido em terços (maior, intermediário, menor); o terceiro terço correspondia aos domicílios mais ricos e o primeiro terço aos domicílios mais pobres.

No acompanhamento aos cinco anos, a insegurança alimentar domiciliar referente aos três meses anteriores à entrevista foi avaliada por meio da versão curta validada da Escala Brasileira de Insegurança Alimentar^19^. No total, cinco questões investigaram se havia preocupação com a escassez de alimentos no domicílio, bem como se a alimentação não era saudável e variada ou se era necessário pular refeições ou reduzir a quantidade de alimentos consumidos por insuficiência de recursos no domicílio. A resposta positiva a qualquer uma das questões foi considerada indicativa de insegurança alimentar.

### Análise Estatística

As características ao nascimento e na infância entre os participantes foram descritas segundo medianas e intervalos interquartis (IIQ) ou proporções. Do nascimento aos cinco anos de idade, os valores das medianas e proporções de interesse dos índices antropométricos foram comparados usando os testes de Wilcoxon e de McNemar (para dados pareados).

Estimou-se a autocorrelação do crescimento linear e do ganho de peso do nascimento até a infância usando modelos de regressão quantílica separadamente para HAZ e WAZ. Realizou-se a regressão das medidas aos cinco anos de idade sobre os escores z de comprimento e peso ao nascer para idade gestacional, respectivamente. Coeficientes e intervalos de confiança de 95% (IC95%) foram extraídos nos quantis 25, 50 e 75 das distribuições de HAZ e WAZ aos cinco anos de idade para explorar o padrão de autocorrelação ao longo das distribuições. Os modelos foram ajustados para a idade exata da criança no acompanhamento aos cinco anos.

Os escores z foram derivados de padrões internacionais que compartilham uma abordagem prescritiva para o crescimento intrauterino e para o crescimento infantil no desenvolvimento das respectivas curvas^11,12^. Os coeficientes das regressões quantílicas podem ser interpretados como coeficientes de correlação^20^ ou como a estabilidade da adequação do estado nutricional exibida entre o par de cada um dos índices antropométricos em diferentes quantis de HAZ e WAZ aos cinco anos de idade. Embora os escores z fossem específicos por sexo, também foi investigada a possibilidade de autocorrelação diferencial, incluindo um termo de interação de sexo com escores z de comprimento e peso ao nascer.

Em uma análise de subgrupo, focou-se em participantes que foram acompanhados aos cinco anos de idade e tinham HAZ e WAZ disponíveis aos dois anos de idade (90% da amostra analítica total, que apresentava características sociodemográficas e perinatais semelhantes). Entre estas crianças, estimaram-se coeficientes de autocorrelação do crescimento linear e do ganho de peso entre o nascimento e os dois anos e entre os dois e os cinco anos de idade. Estimativas (IC95%) também foram extraídas nos quantis 25, 50 e 75 das distribuições de HAZ e WAZ, com ajuste para a idade do participante, respectivamente nas idades de dois e cinco anos. Gráficos de dispersão foram utilizados para comparar visualmente a magnitude da autocorrelação entre esses intervalos de idade.

Por fim, para investigar disparidades na estabilidade do crescimento linear e ganho de peso do nascimento até a infância, foram utilizados modelos de regressão quantílica nos quantis 25, 50 e 75, incluindo variáveis socioeconômicas de interesse e seu termo de interação com escores z de comprimento e peso ao nascer. Ao todo, três variáveis socioeconômicas foram consideradas em modelos distintos: riqueza domiciliar ao nascer (terços); riqueza domiciliar na infância (terços); e insegurança alimentar na infância (sim ou não). O maior nível de riqueza familiar e a ausência de insegurança alimentar foram considerados como categorias de referência. Posteriormente, foram realizadas análises estratificadas para estimar os coeficientes de autocorrelação de HAZ e WAZ (IC95%) segundo categorias das variáveis socioeconômicas. Os valores de p para diferença referem-se aos termos de interação. O nível de significância estatística adotado foi de 5%. Todas as análises foram realizadas no programa Stata, versão 15.1 (StataCorp, College Station, TX).

## RESULTADOS

Das 695 crianças entrevistadas no acompanhamento aos cinco anos, 628 foram elegíveis para as análises, com informações completas sobre desfechos e exposições de interesse disponíveis para mais de 97,5% delas. As crianças com dados completos não diferiram significantemente daquelas que foram acompanhadas e atenderam aos critérios de elegibilidade para esta análise. Ao nascimento, a mediana (IIQ) dos escores z para a idade gestacional foi de 0,06 (−0,58 a 0,86) para o comprimento e de 0,18 (−0,48 a 0,87) para o peso (Tabela 1). Durante o seguimento, não houve variação significante no HAZ (mediana aos 5 anos: 0,06, IIQ: −0,67 a 0,78; p = 0,27) e WAZ (mediana aos 5 anos: 0,17, IIQ: −0,59 a 1,10; p = 0,12). A ocorrência de baixa estatura manteve-se estável (nascimento, 2,1% *versus* 5 anos de idade, 2,3%; p = 0,85), mas aumentos significantes foram observados para baixo peso (nascimento, 0,6% *versus* 5 anos de idade, 2,6%; p = 0,008) e sobrepeso (nascimento, 3,6% *versus* 5 anos de idade, 12,7%; p < 0,001).

**Table 1:** Características ao nascimento e na infância entre participantes do Estudo MINA-Brasil.

Característica		n	Mediana (IIQ) ou %
Sexo			
	Feminino	297	48,4
	Masculino	317	51,6
Idade gestacional ao nascimento (semanas)		614	39,7 (39, 40)
Comprimento ao nascer (cm)		613	49 (48, 51)
Comprimento ao nascer para a idade gestacional (escore z)^a^		613	0,06 (−0,58 a 0,86)
Classificação do comprimento ao nascer^a^			
	Escores z < −2	600	97,9
	Escores z ≥ −2	13	2,1
Peso ao nascer (kg)		614	3,31 (3,05 a 3,62)
Peso ao nascer para a idade gestacional (escore z)^a^		614	0,18 (−0,48 a 0,87)
Classificação do peso ao nascer^a^			
	Escores z < −2	4	0,6
	Escores z ≥ −2 e ≤ +2	588	95,8
	Escores z > +2	22	3,6
Riqueza domiciliar ao nascer			
	Maior	286	46,6
	Intermediário	230	37,4
	Menor	98	16,0
Idade na infância (anos)		614	5,2 (5,1 a 5,4)
Estatura na infância (m)		613	1,12 (1,08 a 1,15)
Estatura para a idade na infância (escore z)^b^		613	0,06 (−0,67 a 0,78)
Classificação da estatura na infância^b^			
	Escores z < −2	599	97,7
	Escores z ≥ −2	14	2,3
Peso na infância (kg)		614	19,4 (17,4 a 22,2)
Peso para idade na infância (escore z)^b^		614	0,17 (−0,59 a 1,10)
Classificação do peso na infância^b^			
	Escores z < −2	16	2,6
	Escores z ≥ −2 e ≤ +2	520	84,7
	Escores z > +2	78	12,7
Riqueza domiciliar na infância			
	Maior	206	33,5
	Intermediário	205	33,4
	Menor	203	33,1
Insegurança alimentar na infância			
	Não	287	46,7
	Sim	327	53,3

Nota: o número de observações pode variar devido a observações faltantes.

a Calculado de acordo com os padrões do Projeto INTERGROWTH-21^st^ para o tamanho do recém-nascido segundo idade gestacional e sexo.

b Calculado de acordo com os Padrões de Crescimento Infantil da OMS segundo idade e sexo.

Como mostrado na Tabela 2, a autocorrelação do crescimento linear do nascimento até a infância foi significante, com magnitude semelhante em toda a distribuição de HAZ aos cinco anos de idade (por exemplo, quantil 50: 0,23, IC95%: 0,11 a 0,35). No geral, a autocorrelação do ganho de peso do nascimento até os cinco anos foi significante e também teve um padrão equivalente em toda a distribuição de WAZ, atingindo 0,38 (IC95%: 0,19 a 0,56) no quantil 75. Não houve evidência de autocorrelação diferencial de acordo com o sexo (p para interação > 0,30 para HAZ e WAZ).

**Table 2:** Autocorrelação do crescimento linear e do ganho de peso do nascimento até a infância no Estudo MINA-Brasil.

Característica		n	Coeficientes de autocorrelação e intervalos de confiança de 95% ao longo da distribuição^a^,^b^		
			Quantil 25	Quantil 50	Quantil 75
Nascimento até 5 anos					
	HAZ	612	0,17 (0,07 a 0,27)	0,23 (0,11 a 0,35)	0,23 (0,13 a 0,33)
	WAZ	614	0,33 (0,21 a 0,45)	0,31 (0,19 a 0,43)	0,38 (0,19 a 0,56)
Nascimento até 2 anos					
	HAZ	551	0,23 (0,12 a 0,34)	0,20 (0,10 a 0,29)	0,28 (0,16 a 0,40)
	WAZ	550	0,41 (0,29 a 0,53)	0,32 (0,21 a 0,43)	0,35 (0,21 a 0,48)
2 a 5 anos					
	HAZ	550	0,82 (0,75 a 0,89)	0,88 (0,82 a 0,94)	0,89 (0,84 a 0,94)
	WAZ	550	0,86 (0,80 a 0,92)	0,96 (0,89 a 1,03)	1,10 (1,00 a 1,20)

HAZ: escore z de comprimento/estatura para idade; WAZ: escore z de peso para idade.

a Os índices antropométricos foram calculados ao nascer de acordo com os padrões do Projeto INTERGROWTH-21^st^ para o tamanho do recém-nascido segundo idade gestacional e sexo e nas idades de dois e cinco anos de acordo com os Padrões de Crescimento Infantil da OMS segundo idade e sexo.

b A autocorrelação foi estimada usando modelos de regressão quantílica separadamente para HAZ e WAZ. Realizou-se a regressão dos escores Z em idade mais avançada sobre aqueles de idade mais precoce, extraindo-se coeficientes e intervalos de confiança de 95% nos quantis 25, 50 e 75.

Em comparação com o período do nascimento até cinco anos, todos os coeficientes de autocorrelação até os dois anos de idade apresentaram magnitude comparável (Figuras 2A e 2B), sem variação entre os quantis (Tabela 2). No entanto, uma autocorrelação marcadamente maior foi observada dos dois aos cinco anos de idade em todos os quantis para o crescimento linear (quantil 50: 0,88, IC95%: 0,82 a 0,94) e ganho de peso (quantil 50: 0,96, IC95%: 0,89 a 1,03), conforme ilustrado nas Figuras 2C e 2D.

**Figura 2:** Dispersão dos escores z de comprimento/estatura e peso ao nascimento e nas idades de dois e cinco anos entre os participantes do Estudo MINA-Brasil.

Nota: Painéis A e B: medidas aos dois anos (pontos pretos) e cinco anos (pontos cinzas) em relação às medidas ao nascimento. Painéis C e D: medidas aos cinco anos de idade em relação às medidas aos dois anos (pontos pretos). A linha tracejada vermelha indica o escore z de comprimento/altura < −2 (Painéis A e C) e o escore z de peso > +2 (Painéis B e D). Os índices antropométricos foram calculados ao nascer de acordo com os padrões do Projeto INTERGROWTH-21^st^ para o tamanho do recém-nascido segundo idade gestacional e sexo, e nas idades de dois e cinco anos de acordo com os Padrões de Crescimento Infantil da OMS segundo idade e sexo.

Houve disparidades na autocorrelação do crescimento linear segundo variáveis socioeconômicas (Tabela 3). Autocorrelação significantemente maior foi observada nos limites inferiores da distribuição de HAZ aos cinco anos de idade entre as crianças expostas a maior riqueza domiciliar ao nascer (quantil 25: 0,30, IC95%: 0,13 a 0,56; quantil 50: 0,38, IC95%: 0,21 a 0,55; valor de p para diferença < 0,05). Em todos os quantis da distribuição de HAZ aos cinco anos de idade, a autocorrelação do crescimento linear desde o nascimento foi mais forte para as crianças dos domicílios mais ricos na infância, em relação àquelas dos domicílios mais pobres, entre as quais os coeficientes não foram significantes (por exemplo, quantil 50: 0,37, IC95%: 0,21 a 0,53 *versus* 0,11, IC95%: −0,07 a 0,28; valor de p para diferença = 0,012). No quantil 75 da distribuição de HAZ aos cinco anos de idade, houve uma diferença marginal na autocorrelação do crescimento linear de acordo com a insegurança alimentar domiciliar na infância (não expostos: 0,32, IC95%: 0,16 a 0,48; expostos: 0,12, 95%CI: 0,00 a 0,24; valor de p para diferença = 0,047).

**Table 3:** Autocorrelação do crescimento linear e ganho de peso do nascimento até a infância segundo grupos socioeconômicos no Estudo MINA-Brasil.

Característica			Coeficientes de autocorrelação ao longo da distribuição aos cinco anos^a^					
			Quantil 25		Quantil 50		Quantil 75	
			Coef. (IC95%)^b^	p dif^c^	Coef. (IC95%)^b^	p dif^c^	Coef. (IC95%)^b^	p dif^c^
Escore z de estatura para idade (HAZ)								
	Riqueza domiciliar ao nascer							
		Maior	0,30 (0,13 a 0,56)	-	0,38 (0,21 a 0,55)	-	0,30 (0,16 a 0,45)	-
		Intermediária	0,04 (−0,10 a 0,18)	0,007	0,12 (−0,02 a 0,26)	0,022	0,10 (−0,11 a 0,31)	0,198
		Menor	0,11 (−0,19 a 0,41)	0,141	0,16 (−0,14 a 0,46)	0,103	0,12 (−0,09 a 0,32)	0,255
	Riqueza domiciliar na infância							
		Maior	0,41 (0,18 a 0,64)	-	0,37 (0,21 a 0,53)	-	0,35 (0,16 a 0,53)	-
		Intermediária	0,04 (−0,14 a 0,21)	0,003	0,22 (0,03 a 0,40)	0,194	0,20 (−0,02 a 0,41)	0,189
		Menor	0,09 (−0,10 a 0,27)	0,010	0,11 (−0,07 a 0,28)	0,012	0,10 (−0,04 a 0,25)	0,014
	Insegurança alimentar na infância							
		Não	0,13 (−0,03 a 0,30)	-	0,34 (0,18 a 0,49)	-	0,32 (0,16 a 0,48)	-
		Sim	0,13 (0,01 a 0,25)	0,689	0,20 (0,06 a 0,35)	0,387	0,12 (0,00 a 0,24)	0,047
Escore z de peso/idade (WAZ)								
	Riqueza domiciliar ao nascer							
		Maior	0,42 (0,23 a 0,62)	-	0,44 (0,25 a 0,63)	-	0,59 (0,35 a 0,83)	-
		Intermediária	0,33 (0,16 a 0,50)	0,550	0,20 (0,00 a 0,40)	0,019	0,03 (−0,29 a 0,34)	0,003
		Menor	0,18 (−0,11 a 0,46)	0,192	0,19 (−0,12 a 0,51)	0,032	0,40 (−0,10 a 0,89)	0,112
	Riqueza domiciliar na infância							
		Maior	0,47 (0,24 a 0,70)	-	0,67 (0,46 a 0,86)	-	0,54 (0,15 a 0,93)	-
		Intermediária	0,28 (0,08 a 0,47)	0,192	0,30 (0,11 a 0,49)	0,013	0,35 (0,05 a 0,66)	0,308
		Menor	0,27 (0,08 a 0,46)	0,186	0,14 (−0,08 a 0,35)	<0,001	0,08 (−0,25 a 0,41)	0,031
	Insegurança alimentar na infância							
		Não	0,27 (0,07 a 0,47)	-	0,43 (0,22 a 0,65)	-	0,52 (0,25 a 0,79)	-
		Sim	0,40 (0,26 a 0,54)	0,348	0,30 (0,16 a 0,45)	0,241	0,24 (0,05 a 0,44)	0,060

HAZ: escore z de comprimento/estatura para idade; WAZ: escore z de peso para idade; Coef.: coeficiente; IC95%: intervalo de confiança de 95%; p dif: valor de p para diferença.

a Os índices antropométricos foram calculados ao nascer de acordo com os padrões do Projeto INTERGROWTH-21st para o tamanho do recém-nascido segundo idade gestacional e sexo e aos cinco anos de acordo com os Padrões de Crescimento Infantil da OMS segundo idade e sexo.

b A autocorrelação foi estimada usando modelos de regressão quantílica separadamente para HAZ e WAZ. Realizou-se a regressão dos escores Z aos cinco anos sobre aqueles ao nascer, extraindo-se coeficientes e intervalos de confiança de 95% nos quantis 25, 50 e 75.

c Para investigar disparidades nas variáveis de riqueza domiciliar e insegurança alimentar foram incluídos termos de interação com os escores z ao nascer. Os valores de p para diferença referem-se aos termos de interação. Análises estratificadas foram realizadas para estimar os coeficientes de autocorrelação de HAZ e WAZ segundo categorias das variáveis socioeconômicas.

Para o ganho de peso (Tabela 3), observou-se maior autocorrelação nos quantis 50 e 75 da distribuição de WAZ aos cinco anos de idade entre as crianças dos domicílios mais ricos ao nascer e na infância, em comparação com os participantes de domicílios com renda intermediária e mais baixa. O coeficiente mediano de autocorrelação do ganho de peso do nascimento até a infância foi de 0,44 (IC95%: 0,25 a 0,63) entre os participantes dos domicílios mais ricos ao nascer (em relação a 0,19, IC95%: −0,12 a 0,51 entre aqueles dos domicílios mais pobres ao nascer, valor de p para diferença = 0,032) e atingiu 0,67 (IC95%: 0,46 a 0,86) para as crianças mais ricas na infância (em relação a 0,14, IC95%: −0,08 a 0,35 entre aquelas das famílias mais pobres na infância; valor de p para diferença < 0,001). Não houve evidência de diferenças na autocorrelação do ganho de peso desde o nascimento ao longo da distribuição de WAZ de acordo com a insegurança alimentar aos cinco anos de idade.

## DISCUSSÃO

Utilizando dados coletados prospectivamente de crianças de até cinco anos de idade participantes do Estudo MINA-Brasil, observou-se autocorrelação significante e positiva do crescimento linear e do ganho de peso do nascimento até a infância, com coeficientes de magnitude semelhante ao longo da distribuição dos índices antropométricos. A estabilidade do estado nutricional foi mais acentuada no período de dois a cinco anos de idade. Além disso, houve autocorrelação diferencial de acordo com a exposição a níveis distintos de riqueza domiciliar.

Na população de estudo, o tamanho médio do recém-nascido ao nascer e o estado nutricional aos cinco anos foram próximos aos valores medianos esperados para comprimento/estatura e peso para idade pelos padrões internacionais. Notou-se um ligeiro desvio para a esquerda para os valores contínuos de HAZ até os cinco anos de idade, como representado pelos intervalos interquartis, mas o déficit de estatura permaneceu baixo durante o acompanhamento. Para o peso das crianças, a distribuição tornou-se mais dispersa ao longo do tempo, com menores valores de WAZ correspondendo ao quantil 25 e maiores valores no quantil 75 aos cinco anos de idade. Isso se refletiu em ocorrência significantemente maior de baixo peso e de excesso de peso na infância, caracterizando dinâmicas de má nutrição no Estudo MINA-Brasil.

A autocorrelação do crescimento linear e do ganho de peso foi significante em geral e comparável entre meninas e meninos. Coeficientes de magnitude moderada e com sobreposição dos IC95% variaram de 0,17 a 0,39 do nascimento aos cinco anos de idade e, em uma análise de subgrupo, de 0,20 a 0,40 do nascimento aos dois anos de idade. Em conjunto, estas estimativas quantificam uma influência não desprezível e bastante duradoura das condições de crescimento fetal, resumidas pelas medidas de tamanho ao nascer, sobre toda a distribuição dos índices antropométricos infantis. Isso é corroborado por achados longitudinais sobre a importância do período gestacional na prevenção do crescimento subótimo e no reconhecimento da necessidade de ações adicionais no início da vida para evitar agravos pós-natais ao crescimento saudável em estatura e peso^6^. De acordo com uma revisão recente, medidas eficazes para alavancar o crescimento neonatal e infantil incluem, mas não são limitadas a, aconselhamento sobre aleitamento materno e alimentação complementar; suplementação de múltiplos micronutrientes; planejamento familiar; controle da malária; e intervenções para acesso a água, saneamento e higiene^21^.

A partir dos dois anos de idade, os coeficientes de autocorrelação foram substancialmente maiores ao longo das distribuições de HAZ e WAZ, acima de 0,82. Este resultado se conecta fortemente com a importância dos primeiros 1.000 dias na promoção do desenvolvimento humano. Em uma abordagem complementar à noção de autocorrelação para investigar trajetórias de crescimento, medidas condicionais do tamanho corporal indicaram que crescimento linear mais rápido e ganho de peso até os dois anos de idade foram benéficos para a altura e escolaridade na idade adulta em países de baixa e média renda, além de proteger contra alguns fatores de risco para doenças crônicas^22,23^. Simultaneamente, sabe-se que os declínios nos escores z de comprimento para idade atingem o pico essencialmente no segundo ano de vida^24^, criando um cenário desafiador para crianças em ambientes com menos recursos. Por meio de uma avaliação empregando padrões de crescimento ideais, as estimativas desse estudo sugeriram que, de fato, os níveis de adequação do estado nutricional alcançados após os dois primeiros anos serão fortemente estáveis até os cinco anos de idade. Isso delimita uma menor probabilidade de mudanças espontâneas nos padrões de trajetórias nesse intervalo e reforça a necessidade de intervenções oportunas baseadas em evidências.

As disparidades socioeconômicas tiveram papéis distintos para moldar a estabilidade de HAZ e WAZ. Em relação à estatura aos cinco anos de idade, pertencer a domicílios mais ricos associou-se a uma autocorrelação significantemente mais forte ao longo de toda a distribuição, o que implica melhores possibilidades de manter a influência positiva do crescimento linear intrauterino no período pós-natal. Além disso, a relação pareceu ser mais relevante para crianças nos limites inferiores da distribuição de HAZ, se considerada a riqueza domiciliar ao nascer. Considerando que crianças que apresentam valores de HAZ abaixo da mediana esperada para sua idade estão provavelmente sujeitas a piores influências ambientais ao crescimento, a exposição a uma melhor posição socioeconômica pode ser valiosa. Este achado é consistente com uma meta-análise global que apontou a concentração de baixa estatura entre crianças menores de cinco anos em domicílios pobres, especialmente na América Latina e no Caribe (índice de concentração regional agrupada: −0,22, IC95%: −0,29 a −0,15)^13^. Na análise combinada de coortes de países de baixa e média renda, a pobreza no início da vida no nível domiciliar também teve impacto negativo no crescimento alcançado em períodos mais tardios, durante a adolescência e a idade adulta^25,26^.

Com relação ao WAZ aos cinco anos de idade, coeficientes de autocorrelação mais fortes foram aparentes para crianças mais pesadas, provenientes de domicílios mais ricos. Nas coortes do Young Lives Study, a razão de risco relativo para uma trajetória de alta probabilidade de sobrepeso variou de 3,1 a 5,6 nos quartos de riqueza superiores na Índia, no Peru e no Vietnã^25^. Este resultado também foi coerente com uma revisão sistemática de estudos em países de média renda que encontrou mais associações positivas entre posição socioeconômica e medidas de massa gorda em escolares (78% de 18 estudos com meninas e 63% de 9 estudos com meninos)^27^, em oposição a crianças de países de alta renda, em que associações inversas emergem em várias pesquisas^20,27,28^.

A presente análise ofereceu uma medida quantitativa da estabilidade das trajetórias de crescimento linear e ganho de peso e padronizou medidas antropométricas seriadas de acordo com recomendações prescritivas comuns de curvas internacionais, conectando a interpretação dos processos de crescimento pré e pós-natal de forma original. Os achados evidenciaram a importância da elaboração de programas primários para promover melhores padrões de crescimento desde antes do nascimento, com acesso equitativo e de qualidade de acordo com as características socioeconômicas locais, uma vez que a riqueza domiciliar incorreu em autocorrelação diferencial do estado nutricional.

Algumas limitações devem ser apontadas. Primeiramente, as perdas de seguimento nesta coorte reduziram o tamanho da amostra para esta análise de casos completos. É importante notar que as perdas no acompanhamento se concentraram em crianças de pior nível socioeconômico e as estimativas podem ter sido atenuadas por viés de seleção. Em segundo lugar, as variáveis ao nascimento foram coletadas dos prontuários médicos. Vieses de aferição não devem ser substanciais, uma vez que a equipe do estudo realizou treinamento sobre avaliação antropométrica dos recém-nascidos e também verificou regularmente os equipamentos para mensuração do tamanho ao nascer^14^. Além disso, a concordância das informações sobre a idade gestacional foi considerada satisfatória em comparação com as medidas obtidas diretamente pela equipe do estudo por meio de exames de ultrassonografia durante o período pré-natal^16^. Em terceiro lugar, as informações sobre insegurança alimentar domiciliar estavam disponíveis apenas aos cinco anos de idade, o que impediu verificar associações ao longo do tempo com a autocorrelação do crescimento linear e do ganho de peso. Ademais, mais medidas repetidas permitiriam examinar a autocorrelação em menores intervalos de idade, mas pudemos investigar períodos-chave sensíveis para prevenir a má nutrição infantil.

Em conclusão, observou-se autocorrelação significante do crescimento linear e do ganho de peso do nascimento até os cinco anos de idade em magnitude semelhante ao longo de toda a distribuição de HAZ e WAZ. A estabilidade substancial dos níveis de adequação do estado nutricional emergiu especificamente entre os dois e os cinco anos de idade. Houve autocorrelação diferencial de acordo com a riqueza domiciliar, que deve ser considerada no planejamento de intervenções precoces para a promoção do crescimento saudável.
